# Blue Light Pollution and Ocular Surface Toxicity: p53-Mediated Ferroptosis in Corneal Epithelium Drives Dry Eye Pathogenesis

**DOI:** 10.3390/antiox15081014

**Published:** 2026-08-14

**Authors:** Lingyu Zhang, Yiwen Qian, Jun Jin, Yu Zhang, Zhiliang Wang, Qingjian Li

**Affiliations:** 1 Tianjin Key Laboratory of Retinal Functions and Diseases, Tianjin Branch of National Clinical Research Center for Ocular Disease, Eye Institute and School of Optometry, Tianjin Medical University Eye Hospital, Tianjin 300384, China; zhanglingyu0907@163.com; 2 Department of Ophthalmology, Huashan Hospital, Fudan University, Shanghai 200040, China; qianyiwenqueeny@163.com (Y.Q.); rasia199807@gmail.com (J.J.); beautyashley@126.com (Y.Z.)

**Keywords:** blue light, dry eye, corneal epithelium, ferroptosis, p53

## Abstract

Purpose: This study investigated the molecular mechanisms underlying blue light-induced corneal epithelial toxicity. Methods: C57BL/6J mice were exposed to 460 nm blue light for 1, 2, or 4 weeks, whereas human corneal epithelial (HCE) cells were exposed for 15 or 30 min. RNA-sequencing with bioinformatic analysis identified p53 signaling and ferroptosis as key pathways. p53 knockdown using siRNA and pharmacological inhibition with pifithrin-α (PFT-α) were employed to validate the mechanism in vitro and in vivo. Results: Blue light exposure caused time-dependent corneal epithelial disruption, reduced tear film stability, increased inflammatory cell infiltration, downregulated K12 expression, and upregulated K10 expression. Blue light induced cell death and G1-phase cell-cycle arrest in HCE cells. Ferroptosis hallmarks included intracellular ferrous iron (Fe^2+^) accumulation, reactive oxygen species (ROS) generation, decreased SLC7A11 and GPX4 expression, and increased COX2 expression. RNA sequencing revealed p53 pathway activation as a master regulator of ferroptosis. p53 knockdown reversed blue light-induced SLC7A11/GPX4 suppression and COX2 upregulation. Topical PFT-α administration attenuated corneal epithelial damage, restored K12 expression, suppressed K10 expression, and normalized ferroptosis markers in vivo. Conclusions: Blue light pollution triggers corneal epithelial ferroptosis through p53-mediated SLC7A11/GPX4 axis suppression. Pharmacological inhibition of p53 represents a promising therapeutic strategy for blue light-associated dry eye.

## 1. Introduction

The proliferation of digital displays has fundamentally transformed modern visual ecology, introducing unprecedented levels of blue light exposure to the human eye [1]. Blue light, constituting the high-energy visible spectrum (400–500 nm), penetrates deeply into ocular tissues and has been implicated in various photobiological effects [2]. While the retinal hazards of blue light have been extensively characterized through studies on photochemical damage, the impact on the anterior segment—particularly the cornea—has received considerably less attention despite being the primary interface with environmental light [3]. The cornea, as the outermost ocular tissue, directly absorbs a substantial proportion of incident blue light, positioning it as a critical but understudied target of phototoxicity.

The global prevalence of dry eye disease has reached epidemic proportions, affecting 5–50% of the adult population across different geographic regions, with digital device usage emerging as a significant risk factor [4]. Previous investigations from our laboratory demonstrated that blue light pollution induces dry eye through cAMP-PKA-Pax6 signaling pathway-mediated damage to conjunctival stem cells [1]. However, the direct effects of blue light on corneal epithelium—the primary refractive and barrier structure of the ocular surface—remain incompletely characterized at the molecular level.

The corneal epithelium constitutes a stratified squamous epithelium maintained through tight regulation of proliferation, differentiation, and cell death [5]. Keratin 12 (K12) serves as the definitive differentiation marker for corneal-type epithelial cells, while aberrant expression of keratin 10 (K10) indicates pathological squamous metaplasia [6]. Maintenance of corneal epithelial homeostasis requires precise coordination of cell-cycle progression, with disruptions leading to compromised barrier function and increased susceptibility to environmental insults. The high metabolic activity and constant exposure to oxidative stress render corneal epithelial cells particularly vulnerable to photodamage [7].

Ferroptosis, an iron-dependent form of regulated cell death, has emerged as a critical mechanism in various ocular pathologies including age-related macular degeneration [8] and cataract [9]. Unlike apoptosis, necrosis, and autophagy, ferroptosis is characterized by intracellular iron accumulation, lipid peroxidation, and depletion of glutathione (GSH) [10]. The cystine/glutamate antiporter system Xc^−^, composed of solute carrier family 7 member 11 (SLC7A11) and solute carrier family 3 member 2 (SLC3A2), facilitates cystine import essential for GSH synthesis. Glutathione peroxidase 4 (GPX4) subsequently utilizes GSH to reduce lipid hydroperoxides, protecting cellular membranes from oxidative damage. Disruption of this antioxidant axis—through SLC7A11 inhibition or GPX4 inactivation—triggers ferroptotic cell death [11]. Cyclooxygenase-2 (COX2) has been established as a transcriptional marker of ferroptosis, with its upregulation correlating with ferroptotic activity [12].

The tumor suppressor protein p53 orchestrates cellular responses to diverse stress signals including DNA damage, oxidative stress, and oncogenic activation. Beyond its canonical roles in cell-cycle arrest and apoptosis, p53 has been increasingly recognized as a modulator of ferroptosis [13]. Under conditions of severe oxidative stress or DNA damage, activated p53 promotes ferroptotic cell death through multiple mechanisms. Notably, p53 transcriptionally represses SLC7A11, thereby depleting intracellular GSH and sensitizing cells to lipid peroxidation [14]. Additionally, p53 can induce the expression of SAT1, which promotes polyamine metabolism and subsequent lipid peroxidation, further facilitating ferroptosis [15].

Despite growing recognition of blue light as an environmental stressor, the molecular mechanisms linking blue light exposure to corneal epithelial dysfunction remain poorly defined. Previous studies have focused predominantly on oxidative stress or apoptotic pathways [7,16,17]. The involvement of ferroptosis in blue light-induced corneal damage has not been previously explored, and the potential role of p53 as a master regulator of this process represents a significant knowledge gap. Understanding these mechanisms carries important clinical implications, as dry eye currently lacks mechanism-based clinical therapies that address the fundamental causes of epithelial disruption.

Herein, we present comprehensive in vivo and in vitro evidence establishing that blue light exposure induces corneal epithelial ferroptosis through p53-mediated suppression of the SLC7A11/GPX4 antioxidant axis. Using a clinically relevant mouse model of blue light exposure and human corneal epithelial (HCE) cells, we demonstrate time-dependent epithelial disruption, inflammatory cell infiltration, and molecular alterations characteristic of ferroptosis. Transcriptomic analysis identified p53 signaling pathway activation as the upstream regulator, with subsequent validation through genetic and pharmacological interventions confirming the therapeutic potential of p53 inhibition. Our findings establish a mechanistic framework linking blue light pollution to corneal epithelial toxicity and identify p53-targeted ferroptosis inhibition as a promising strategy for preventing and treating blue light-associated dry eye.

## 2. Materials and Methods

### 2.1. Experimental Cell Culture System

The immortalized HCE cell line (CRL-3582, American Type Culture Collection, Manassas, VA, USA) was propagated in DMEM/F12 medium (C11330500BT, Thermo Fisher Scientific, Waltham, MA, USA) supplemented with 5 μg/mL human recombinant epidermal growth factor (PHG0311, Thermo Fisher Scientific), 5 μg/mL insulin (PB180431, Procell, Wuhan, China), 10% fetal bovine serum (AB-FBS-1050, ABW, Shanghai, China), and 100 U/mL penicillin-streptomycin (15070063, Thermo Fisher Scientific). Cultures were maintained at 37 °C in a humidified atmosphere containing 5% CO_2_, with medium renewal every 48 h. Cells in the logarithmic growth phase were utilized for all experimental procedures, with passage numbers between 3 and 8 to ensure consistent phenotypic characteristics.

### 2.2. Animal Maintenance and Ethical Compliance

All animal procedures conformed to the Association for Research in Vision and Ophthalmology Statement for the Use of Animals in Ophthalmic and Vision Research and received formal approval from the Institutional Animal Care and Use Committee of Fudan University (Protocol Number: 2024-HSYY-566). Male C57BL/6J mice aged 7–8 weeks were accommodated in a specific pathogen-free facility with controlled environmental parameters: ambient temperature maintained at 25 °C, relative humidity of 50–60%, and a 12 h light/dark cycle. Animals had unrestricted access to sterilized food and autoclaved water throughout the experimental period.

### 2.3. Blue Light Irradiation Protocol

Custom-designed blue light-emitting devices were engineered to deliver uniform illumination at 460 nm wavelength, corresponding to the peak emission spectrum of digital displays. Spectral irradiance measurements were performed using a spectral illuminance analyzer (Hopoocolor, Hangzhou, China) to ensure consistent output. The illumination intensity was standardized at 1000 lux, as previously described [1,18]. Environmental temperature was maintained at 25 °C throughout exposure periods to exclude thermal effects.

For in vivo experiments, mice were exposed to blue light for 8 consecutive hours daily over durations of 1, 2, or 4 weeks. Age-matched control animals were maintained under identical conditions without blue light exposure. For in vitro studies, HCE cells seeded in culture dishes were exposed to blue light for 15 or 30 min at 37 °C, with non-irradiated cells serving as controls.

### 2.4. Assessment of Cellular Viability and Morphological Alterations

Cell viability was quantitatively evaluated using the Cell Counting Kit-8 (CCK-8) assay (ABclonal, Wuhan, China). Following experimental treatments, HCE cells cultured in 96-well plates were incubated with 10% CCK-8 reagent diluted in culture medium for 3 h at 37 °C. Absorbance at 450 nm was measured using a microplate reader (Thermo Fisher Scientific). HCE cells were examined under an inverted phase-contrast microscope (Leica Microsystems, Wetzlar, Germany) to document morphological changes, with representative images captured from randomly selected fields.

### 2.5. Histopathological Evaluation

Mouse eyeballs were fixed in 4% paraformaldehyde solution (Servicebio, Wuhan, China) at 4 °C for 24 h, followed by paraffin embedding using standard histological procedures. Serial sections of 5 μm thickness were prepared using a rotary microtome (Leica Biosystems, Nussloch, Germany). For hematoxylin and eosin staining, sections were deparaffinized in xylene, rehydrated through graded ethanol concentrations, and stained with hematoxylin for 5 min followed by eosin for 2 min. Stained sections were dehydrated, cleared, mounted with neutral resin, and examined under a light microscope (Leica Microsystems). Corneal epithelial thickness measurements were performed on digital images using ImageJ software (version 1.54p, National Institutes of Health, Bethesda, MD, USA) at central and peripheral regions.

### 2.6. Ocular Surface Evaluation

Corneal epithelial integrity was assessed by instilling 10 μL of 1% sodium fluorescein solution into the inferior conjunctival sac. After 15 s, eyes were gently rinsed with physiological saline to remove excess dye, and corneal staining was photographed using a slit-lamp biomicroscope equipped with cobalt blue illumination (Keeler, Windsor, UK). Fluorescein staining intensity was graded according to the National Eye Institute scoring system.

Tear film stability was evaluated by measuring tear break-up time (TBUT). Following fluorescein instillation, mice were manually blinked to distribute the tear film, and the time elapsed until the appearance of the first dry spot on the corneal surface was recorded using stopwatch timing. Three consecutive measurements were performed for each eye and averaged.

### 2.7. In Vivo Confocal Microscopy

Corneal inflammatory cell infiltration was examined using the Heidelberg Retina Tomograph III with Rostock Cornea Module (Heidelberg Engineering, Heidelberg, Germany). Mice were anesthetized and positioned on the microscope stage with the cornea applanated against the sterile TomoCap (Heidelberg Engineering). Sequential images were acquired from the superficial epithelium through the deep stroma. Inflammatory cells were identified as hyperreflective punctate structures with characteristic morphological features.

### 2.8. Optical Coherence Tomography

Corneal thickness measurements were performed using an OPTOPROBE system (OPTOPROBE, Beijing, China). Anesthetized mice were positioned with the cornea perpendicular to the scanning beam. High-resolution cross-sectional images through the central cornea were acquired, and corneal thickness was measured at the apex using caliper tools provided by the instrument software.

### 2.9. Immunoblotting Analysis

Cultured HCE cells or dissected corneal tissues were lysed in RIPA buffer (Thermo Fisher Scientific) supplemented with Halt Protease and Phosphatase Inhibitor Cocktail (Thermo Fisher Scientific). Following centrifugation at 12,000× *g* for 15 min at 4 °C, supernatant protein concentrations were determined using the BCA Protein Assay Kit (Thermo Fisher Scientific). Equal protein amounts (15 μg per lane) were separated by electrophoresis on 10% SDS-polyacrylamide gels and transferred electrophoretically to polyvinylidene difluoride membranes (Roche, Basel, Switzerland). Membranes were blocked with 5% bovine serum albumin in Tris-buffered saline containing 0.05% Tween-20 for 1 h at ambient temperature, followed by overnight incubation at 4 °C with primary antibodies diluted 1:1000 in blocking buffer. Primary antibodies included rabbit anti-SLC7A11 (A2413, ABclonal), mouse anti-GPX4 (67763-1-Ig, Proteintech, Rosemont, IL, USA), rabbit anti-COX2 (12282S, Cell Signaling Technology, Danvers, MA, USA), rabbit anti-p53 (10442-1-AP, Proteintech), rabbit anti-K12 (ab185627, Abcam, Cambridge, UK), and rabbit anti-K10 (ab76318, Abcam). After three washes with TBST, membranes were incubated with horseradish peroxidase-conjugated goat anti-mouse (31430, Thermo Fisher Scientific) or goat anti-rabbit (31460, Thermo Fisher Scientific) secondary antibodies diluted 1:5000 for 1 h at room temperature. HRP-conjugated mouse anti-β-actin antibody (1:2000, Servicebio) served as a loading control. Protein bands were visualized using enhanced chemiluminescence substrate (NCM Biotech, Suzhou, China) and imaged with the ChemiDoc XRS System (Bio-Rad Laboratories, Hercules, CA, USA). Densitometric analysis was performed using Image Lab software (version 5.2, Bio-Rad).

### 2.10. RNA Isolation and Quantitative Real-Time PCR

Total RNA was extracted from HCE cells or corneal tissues using TRIzol reagent (Thermo Fisher Scientific) according to the manufacturer’s protocol. RNA concentration and purity were assessed spectrophotometrically using NanoDrop One (Thermo Fisher Scientific), with A260/A280 ratios between 1.8 and 2.0 considered acceptable. Complementary DNA was synthesized from 1 μg total RNA using PrimeScript RT Master Mix (TaKaRa, Shiga, Japan) in a 20 μL reaction volume. Quantitative Real-Time PCR (qRT-PCR) was performed on a LightCycler 96 Instrument (Roche) using Taq Pro Universal SYBR qPCR Master Mix (Vazyme, Nanjing, China). Each 10 μL reaction contained 5 μL SYBR Master Mix, 0.2 μL each of forward and reverse primers (10 μM), 1 μL cDNA template, and 3.6 μL RNase-free water. Thermal cycling conditions comprised initial denaturation at 95 °C for 30 s, followed by 40 cycles of 95 °C for 10 s, 60 °C for 20 s, and 72 °C for 20 s. Melting curve analysis was performed to verify amplification specificity. Relative gene expression was calculated using the 2^−ΔΔCt^ method with β-actin as the endogenous reference. Primer sequences are presented in Table 1.

### 2.11. Intracellular Reactive Oxygen Species Detection

Intracellular reactive oxygen species (ROS) levels were assessed using the fluorescent probe 2′,7′-dichlorodihydrofluorescein diacetate (DCFH-DA; Beyotime, Shanghai, China). Following blue light exposure, HCE cells were incubated with 10 μM DCFH-DA in serum-free medium for 20 min at 37 °C in the dark. After three washes with pre-warmed PBS, fluorescence intensity was quantified using a CytoFLEX flow cytometer (Beckman Coulter, Brea, CA, USA) with excitation at 488 nm and emission detection at 525 nm. For microscopic visualization, cells were co-stained with 10 μM Hoechst 33342 (Sigma-Aldrich, St. Louis, MO, USA) and examined under a fluorescence microscope (Leica Microsystems). At least 10,000 events were acquired per sample for flow cytometric analysis.

### 2.12. Iron Staining

Intracellular ferrous iron (Fe^2+^) was detected using FerroOrange probe (Dojindo Laboratories, Kumamoto, Japan) according to manufacturer specifications. HCE cells cultured on glass coverslips were washed three times with Hank’s Balanced Salt Solution (Gibco, Carlsbad, CA, USA) and incubated with 1 μM FerroOrange and 10 μM Hoechst 33342 for 30 min at 37 °C in the dark. After washing, coverslips were mounted on glass slides and immediately imaged using a fluorescence microscope equipped with appropriate filter sets (excitation 543 nm, emission 580 nm for FerroOrange; excitation 350 nm, emission 461 nm for Hoechst).

### 2.13. Cell-Cycle Flow Cytometry Analysis

HCE cells in the logarithmic growth phase were seeded into 6-well plates at a density of 7000 cells/cm^2^. Upon reaching approximately 60–70% confluence, the cells were subjected to blue light irradiation. Following the treatment, cells were harvested via trypsinization. The cell suspension was equally aliquoted into six pre-chilled 1.5 mL microcentrifuge tubes according to the experimental groups. Cells were pelleted by centrifugation at 1500 rpm for 5 min, and the culture medium was discarded. The cells were then washed three times with 500 μL of pre-chilled (4 °C) 1×PBS (Servicebio). For fixation, pre-chilled (−20 °C) 70% ethanol was added dropwise to a final volume of 1 mL. After gentle mixing, the cells were dynamically fixed on a horizontal shaker (80 rpm) at 4 °C for 4 h. Post-fixation, the cells were centrifuged at 1500 rpm for 5 min to remove the ethanol, washed once with 1×PBS, and centrifuged again to discard the supernatant. The cell pellet was slowly and thoroughly resuspended in 0.5 mL of propidium iodide (PI) staining solution (Beyotime, Shanghai, China) and incubated at 37 °C for 30 min in the dark. Finally, the stained suspension was filtered through a 200-mesh sieve and analyzed using a CytoFLEX flow cytometer (Beckman Coulter). Red fluorescence and light scattering were detected at an excitation wavelength of 488 nm. At least 10,000 events were acquired per sample for flow cytometric analysis.

### 2.14. RNA Sequencing and Bioinformatics Analysis

HCE cells exposed to blue light for 30 min or maintained under control conditions were harvested in QIAzol Lysis Reagent (Qiagen, Düsseldorf, Germany). RNA integrity was verified using the Agilent 2100 Bioanalyzer (Agilent Technologies, Santa Clara, CA, USA), with RNA integrity numbers exceeding 8.0 considered acceptable for library preparation. Sequencing libraries were constructed using the Illumina Stranded mRNA Prep Ligation kit (Illumina, San Diego, CA, USA) and sequenced on the Illumina NovaSeq Xplus platform with 150 bp paired-end reads, generating approximately 40 million reads per sample. Three biological replicates per condition were analyzed.

### 2.15. siRNA Transfection

Three independent small interfering RNA sequences targeting human p53 were designed and synthesized by Sangon Biotech (Shanghai, China). HCE cells at 40–50% confluence were transfected with siRNA using the Lipofectamine RNAiMAX reagent (Thermo Fisher Scientific) according to manufacturer instructions. Transfection efficiency was assessed by qRT-PCR and immunoblotting. Non-targeting scrambled siRNA served as a negative control.

### 2.16. Pharmacological Inhibition In Vivo

For in vivo validation, a separate cohort of mice underwent blue light exposure for 2 weeks as described. The p53-specific inhibitor pifithrin-α (PFT-α; Beyotime, Shanghai, China) was dissolved in dimethyl sulfoxide and diluted in phosphate-buffered saline to a final concentration of 500 μM. Mice received topical application of 5 μL PFT-α solution or vehicle control to both eyes four times daily throughout the 2-week exposure period. Ocular surface evaluations and tissue collection were performed at the experimental endpoint.

### 2.17. Statistical Analysis

The Flow Cytometry data were analyzed using FlowJo 10.8.1 (BD Biosciences, Ashland, OR, USA). The data were analyzed using GraphPad Prism version 8.0 (GraphPad Software, San Diego, CA, USA). Results are expressed as mean ± standard deviation (SD) from at least three independent experiments unless otherwise specified. Multiple group comparisons were conducted using one-way analysis of variance followed by Tukey’s post hoc test for pairwise comparisons. Statistical significance was defined as *p* < 0.05.

## 3. Results

### 3.1. Blue Light Exposure Induces Progressive Ocular Surface Disruption and Inflammatory Infiltration

**Figure 1:**
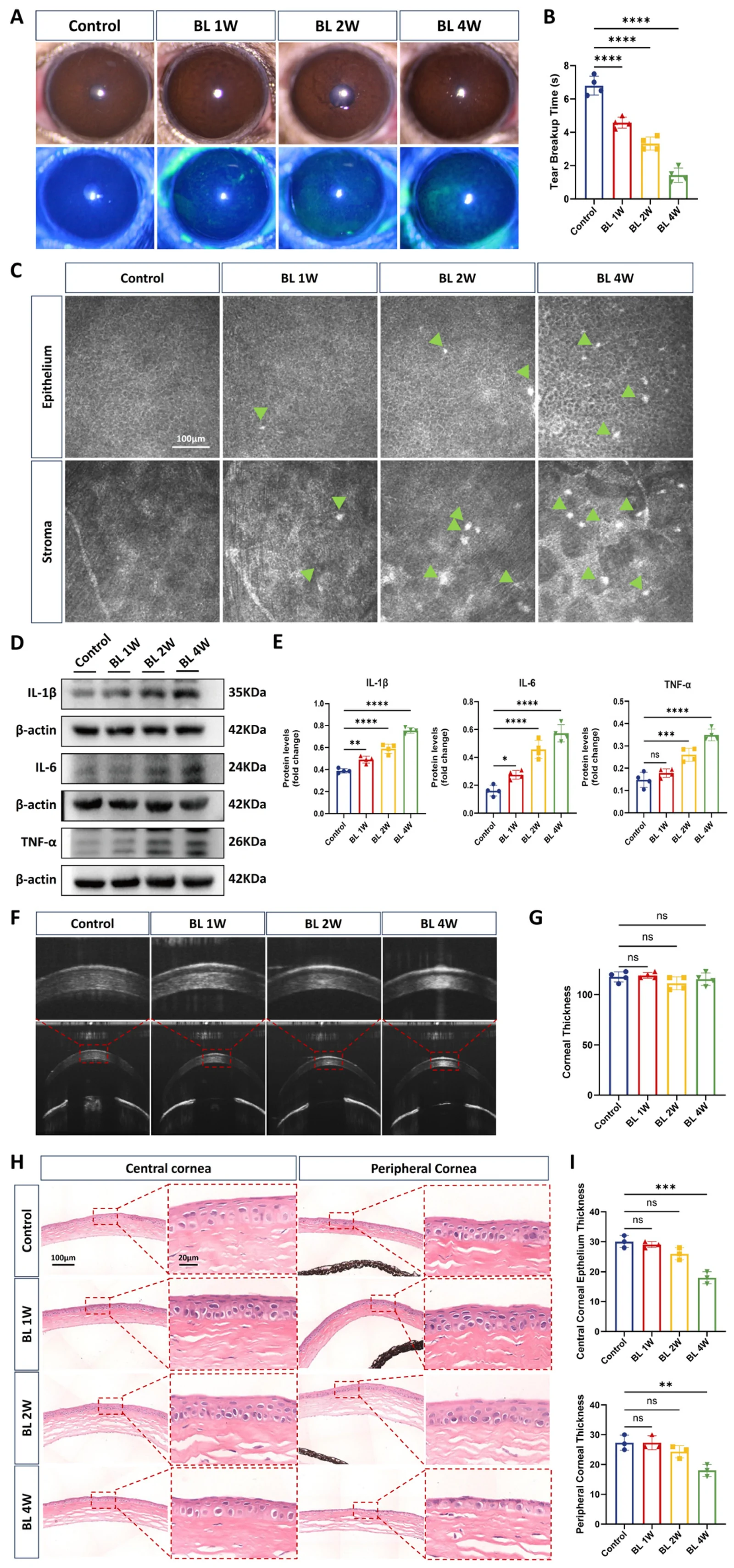
Blue light exposure induces time-dependent ocular surface damage and inflammatory infiltration in mice. Mice were exposed to 460 nm blue light (1000 lux, 8 h/day) for 1, 2, or 4 weeks. (**A**) Representative slit-lamp photographs after corneal fluorescein staining show progressive epithelial disruption with increasing exposure duration. (**B**) Tear break-up time (TBUT) measurements indicate progressive tear film instability (*n* = 4). (**C**) In vivo confocal microscopy images reveal accumulation of inflammatory cells (green arrows) in the basal epithelium and stroma following blue light exposure (scale bar = 100 μm). (**D**) Immunoblotting analysis of pro-inflammatory cytokines interleukin (IL)-1β, IL-6, and tumor necrosis factor (TNF)-α in corneal tissues. (**E**) Densitometric quantification confirms time-dependent upregulation of IL-1β, IL-6, and TNF-α protein levels following blue light exposure (*n* = 4). (**F**) Representative optical coherence tomography images of the central cornea. (**G**) Quantitative analysis shows no significant change in central corneal thickness across groups (*n* = 4). (**H**) Hematoxylin and eosin staining of corneal sections (scale bar = 100/20 μm). (**I**) Quantification of central and peripheral corneal epithelial thickness reveals significant thinning at 4 weeks (*n* = 3). Data are presented as mean ± standard deviation (SD). “ns”, not significant; “*”, *p* < 0.05; “**”, *p* < 0.01; “***”, *p* < 0.001; “****”, *p* < 0.0001. One-way ANOVA with Tukey’s post hoc test.

To characterize the effects of blue light on ocular surface integrity, we subjected C57BL/6J mice to daily 8 h exposures to 460 nm blue light at 1000 lux for durations of 1, 2, or 4 weeks. Slit-lamp examination revealed progressive corneal epithelial disruption with increasing exposure duration. Blue light-exposed mice demonstrated time-dependent increases in corneal staining intensity, indicating compromised epithelial barrier function (Figure 1A). Notably, corneal transparency remained preserved across all time points, suggesting that observed effects were confined to the epithelial layer without stromal opacification. Tear film stability, assessed by TBUT measurements, showed progressive deterioration following blue light exposure (Figure 1B). This progressive instability likely reflects the underlying corneal epithelial disruption compromising tear film adhesion.

In vivo confocal microscopy enabled high-resolution examination of cellular-level changes within the cornea. Control corneas displayed regular epithelial architecture with clearly defined cell borders and minimal hyperreflective structures. Following blue light exposure, we observed progressive accumulation of small, bright punctate cells with morphological characteristics consistent with inflammatory infiltrates (Figure 1C, green arrows). These infiltrates were initially detected in the basal epithelial layer at 1 week, becoming increasingly numerous and extending into the stroma at 2 and 4 weeks. To confirm the inflammatory nature of the observed cellular infiltration, we examined the expression of pro-inflammatory cytokines in corneal tissues by immunoblotting. Blue light exposure resulted in a time-dependent upregulation of interleukin (IL)-1β, IL-6, and tumor necrosis factor (TNF)-α protein levels (Figure 1D,E). These molecular findings corroborate the confocal microscopy observations.

Optical coherence tomography was employed to assess potential alterations in corneal thickness. Central corneal thickness measurements revealed no significant differences between control mice and animals exposed for 1 week, 2 weeks, or 4 weeks (Figure 1F,G), indicating that overall corneal architecture remained intact despite surface epithelial disruption.

Histological examination with hematoxylin and eosin staining provided detailed morphological assessment of corneal epithelial structure. Following blue light exposure, we observed time-dependent thinning of the corneal epithelium. Quantitative analysis revealed significant epithelial thinning at 4 weeks compared with controls in both central and peripheral regions (Figure 1H,I). Additionally, basal epithelial cells displayed marked morphological alterations following blue light exposure, appearing smaller and exhibiting irregular orientation. These findings demonstrate that blue light exposure induces progressive corneal epithelial damage characterized by barrier disruption, inflammatory infiltration, and epithelial thinning.

### 3.2. Blue Light Suppresses Corneal Epithelial Cell Viability and Induces Cell-Cycle Arrest

**Figure 2:**
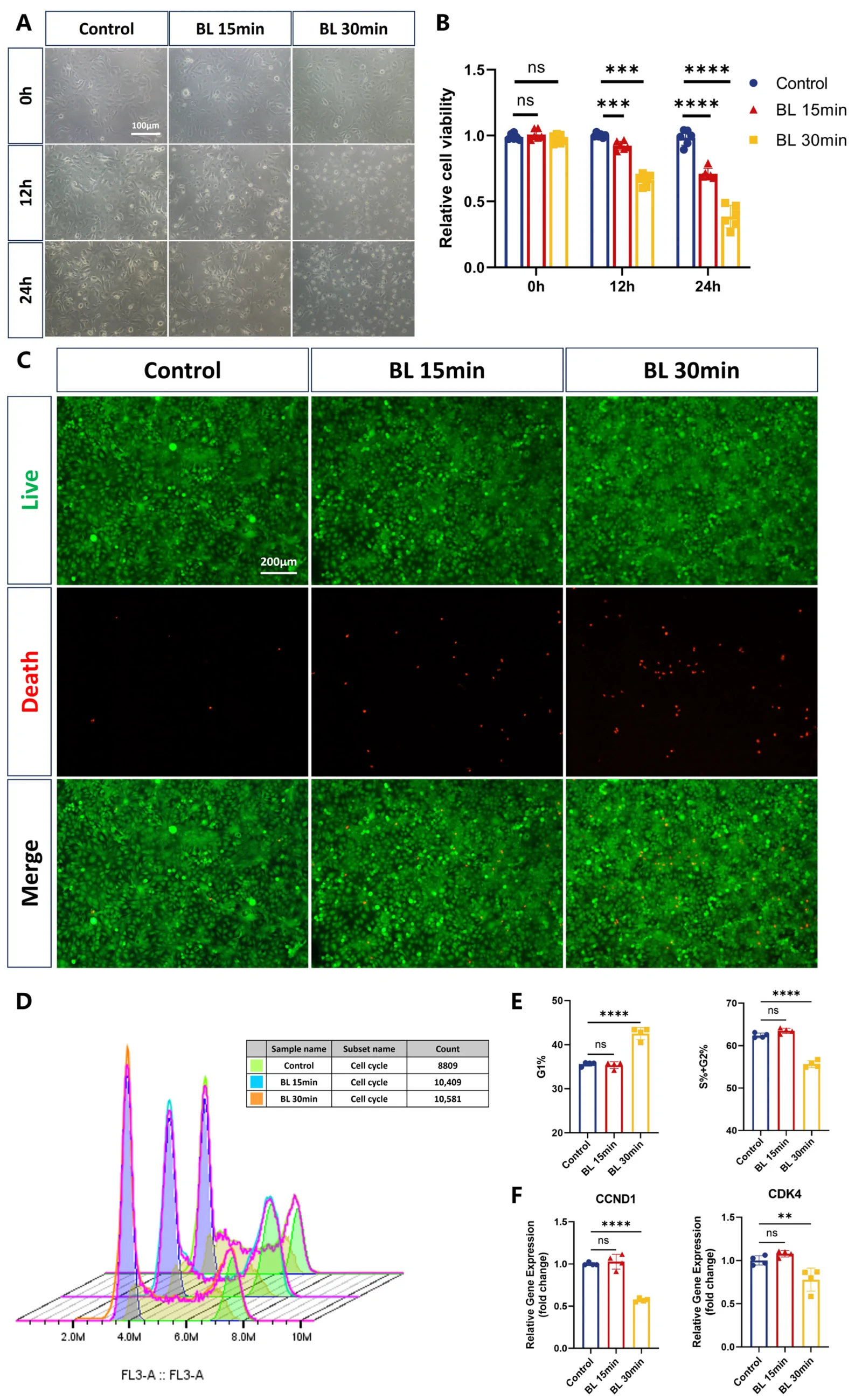
Blue light exposure induces cell death and G1-phase cell-cycle arrest in human corneal epithelial cells. Human corneal epithelial (HCE) cells were exposed to 460 nm blue light for 15 or 30 min. (**A**) Phase-contrast microscopy shows time- and dose-dependent reduction in adherent cells (scale bar = 100 μm). (**B**) Cell Counting Kit-8 (CCK-8) assay demonstrates decreased cell viability in a time-dependent manner (*n* = 6). (**C**) Live–dead staining shows increased dead cells (red) after blue light exposure (scale bar = 200 μm). (**D**,**E**) Flow cytometric analysis reveals G1 phase accumulation following 30 min blue light exposure (*n* = 4). (**F**) Quantitative real-time PCR (qRT-PCR) analysis shows reduced mRNA expression of G1 phase regulators CCND1 and CDK4 (*n* = 4). Data are presented as mean ± SD. “ns”, not significant; “**”, *p* < 0.01; “***”, *p* < 0.001; “****”, *p* < 0.0001. One-way ANOVA with Tukey’s post hoc test.

To elucidate the cellular mechanisms underlying blue light-induced epithelial damage, we established an in vitro model using HCE cells exposed to 460 nm blue light for 15 or 30 min, with subsequent analysis at 12 and 24 h post-exposure. Phase-contrast microscopy revealed progressive loss of adherent cells following blue light irradiation. At 12 h post-exposure, 15 min irradiated cultures showed reduced cell density, while 30 min exposure resulted in more pronounced cell loss and extensive cellular debris (Figure 2A). These changes were further exacerbated at 24 h, with 30 min exposure groups exhibiting near-complete monolayer disruption.

Quantitative assessment of cell viability using CCK-8 assays confirmed the microscopy observations. Compared with control cells, blue light exposure for 15 min reduced viability, and the 30 min exposure produced more severe effects, demonstrating time-dependent cytotoxicity (Figure 2B).

Live–dead staining using calcein-AM and PI provided visual confirmation of blue light-induced cell death. Control cultures showed predominantly green fluorescence with rare red-stained dead cells. Following blue light exposure, the proportion of PI-positive cells increased substantially, with 30 min exposure groups displaying numerous dead cells interspersed among surviving populations (Figure 2C).

Given the observed effects on cell survival, we investigated whether blue light exposure also influenced corneal epithelial proliferation capacity. Flow cytometric cell-cycle analysis revealed that 30 min blue light exposure significantly altered cell-cycle distribution (Figure 2D,E). This G1 phase accumulation indicates cell-cycle arrest, potentially limiting the regenerative capacity required for epithelial maintenance.

To confirm the cell-cycle findings at the molecular level, we examined expression of key G1 phase regulators. qRT-PCR analysis demonstrated that 30 min blue light exposure significantly reduced mRNA levels of cyclin D1 (CCND1) and cyclin-dependent kinase 4 (CDK4) (Figure 2F). These results provide mechanistic validation of the G1 phase arrest observed by flow cytometry and establish that blue light suppresses both cell survival and proliferative capacity of corneal epithelial cells.

### 3.3. Blue Light Induces Keratin Expression Alterations and Ferroptotic Cell Death

**Figure 3:**
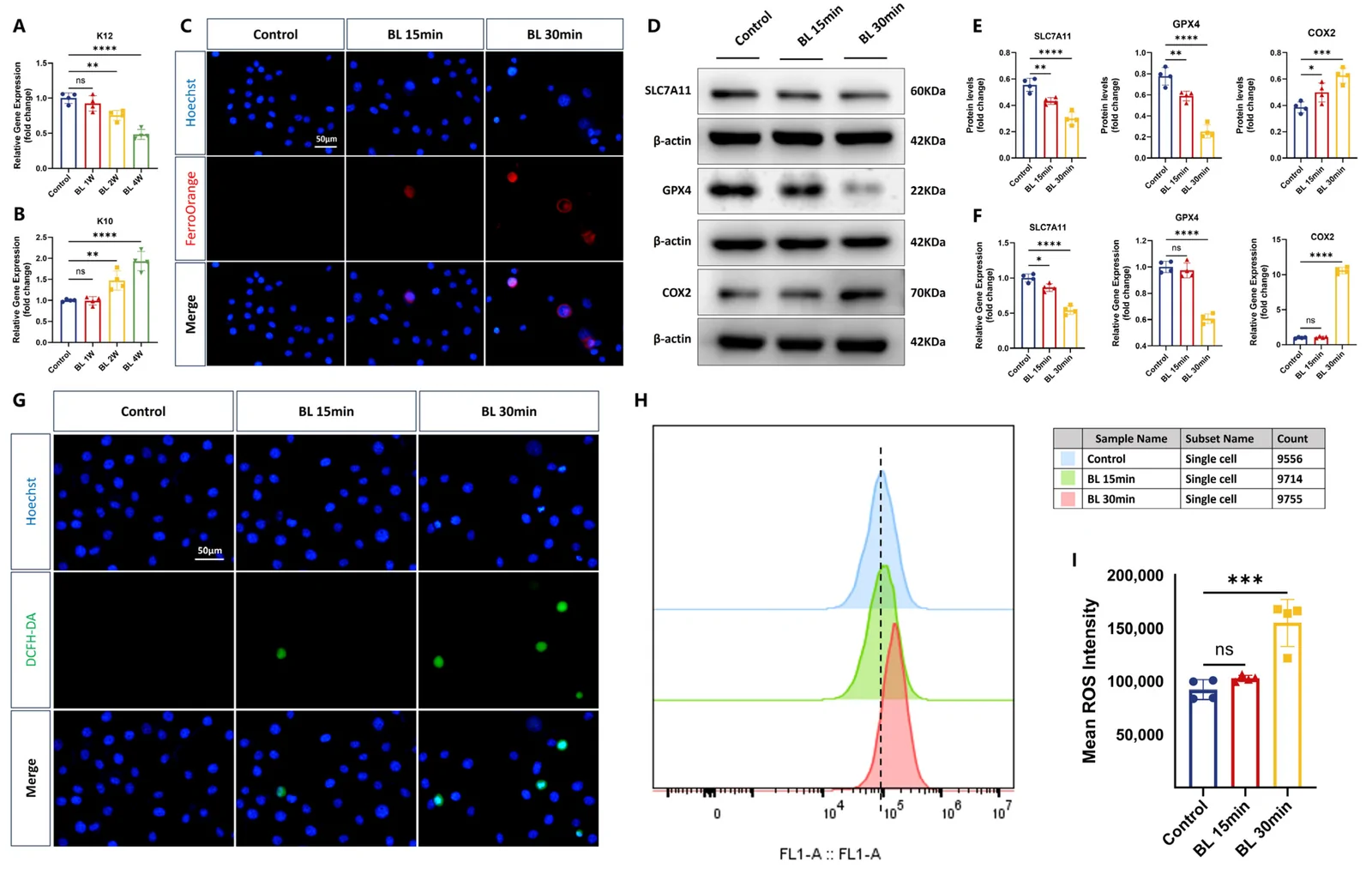
Blue light exposure induces keratin switching and ferroptosis. (**A**,**B**) qRT-PCR analysis shows downregulation of cornea-specific keratin 12 (K12) and upregulation of squamous metaplasia marker keratin 10 (K10) (*n* = 4). (**C**) FerroOrange staining reveals intracellular ferrous iron (Fe^2+^) accumulation after blue light exposure (scale bar = 50 µm). (**D**,**E**) Immunoblotting analysis and densitometric quantification show decreased solute carrier family 7 member 11 (SLC7A11) and glutathione peroxidase 4 (GPX4) protein levels and increased cyclooxygenase-2 (COX2) expression following blue light exposure (*n* = 4). (**F**) qRT-PCR confirms corresponding mRNA changes (*n* = 4). (**G**–**I**) DCFH-DA staining and flow cytometric analysis demonstrate increased reactive oxygen species (ROS) generation after blue light exposure (scale bar = 50 µm; *n* = 4). Data are presented as mean ± SD. “ns”, not significant; “*”, *p* < 0.05; “**”, *p* < 0.01; “***”, *p* < 0.001; “****”, *p* < 0.0001. One-way ANOVA with Tukey’s post hoc test.

The observed morphological changes and cell death prompted investigation into whether blue light exposure affects corneal epithelial cell identity and the specific modality of cell death involved. qRT-PCR analysis of keratin expression revealed significant alterations in differentiation markers following blue light exposure. The cornea-specific K12, essential for maintaining corneal epithelial phenotype, showed progressive downregulation with increasing exposure duration (Figure 3A). Conversely, K10, a marker of squamous metaplasia and pathological epidermal differentiation, exhibited progressive upregulation (Figure 3B). These reciprocal alterations in keratin expression suggest that blue light exposure promotes transdifferentiation toward a non-corneal phenotype, potentially compromising specialized epithelial functions.

Given the iron dependence of light-induced damage in other tissues, we investigated ferroptosis as a potential cell death mechanism. FerroOrange staining for intracellular Fe^2+^ revealed marked accumulation following blue light exposure (Figure 3C).

Immunoblotting analysis examined key regulators of ferroptosis. The cystine/glutamate antiporter subunit SLC7A11, essential for GSH synthesis, showed time-dependent downregulation following blue light exposure. GPX4, the central enzyme neutralizing lipid peroxides, exhibited similar reductions. Conversely, COX2, a transcriptional marker of ferroptosis, demonstrated progressive upregulation (Figure 3D,E). qRT-PCR analysis confirmed these findings at the transcriptional level, with SLC7A11 and GPX4 mRNA showing parallel reductions while COX2 mRNA increased substantially following blue light exposure (Figure 3F).

ROS generation represents a hallmark of ferroptotic cell death. Using DCFH-DA fluorescence, we assessed intracellular ROS accumulation following blue light exposure. Fluorescence microscopy revealed minimal basal ROS in control cells, while blue light-exposed cultures exhibited intense green fluorescence indicating substantial ROS generation. Flow cytometric quantification confirmed these observations (Figure 3G–I). Collectively, these results establish that blue light exposure induces corneal epithelial ferroptosis characterized by iron accumulation, suppression of the SLC7A11/GPX4 antioxidant axis, COX2 upregulation, and ROS generation, accompanied by pathological alterations in keratin expression.

### 3.4. Transcriptomic Analysis Identifies p53 Signaling as Master Regulator of Blue Light-Induced Ferroptosis

**Figure 4:**
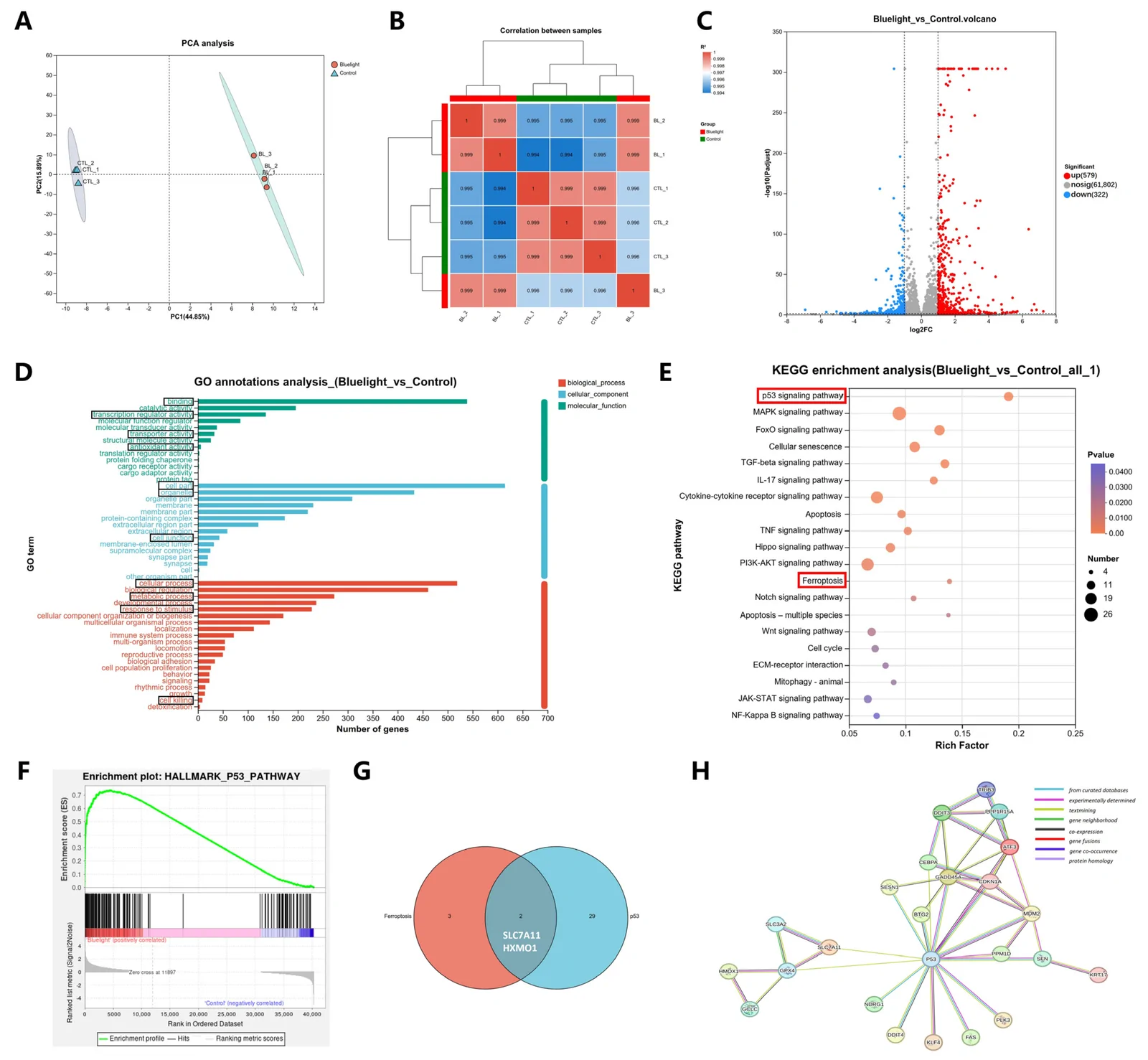
RNA-sequencing analysis identifies p53 signaling as a master regulator of blue light-induced ferroptosis. HCE cells were collected after 30 min of blue light exposure or maintained under control conditions (*n* = 3). (**A**) Principal component analysis shows clear separation between control and blue light-exposed samples. (**B**) Sample correlation heatmap confirms high within-group reproducibility. (**C**) Volcano plot displays differentially expressed genes (|log_2_FC| > 1, adjusted *p* < 0.05), with 579 upregulated and 322 downregulated genes. (**D**) Gene Ontology enrichment analysis reveals alterations in biological process, cellular component, and molecular function categories. (**E**) Kyoto Encyclopedia of Genes and Genomes pathway enrichment analysis identifies the p53 signaling pathway and ferroptosis among the most significantly enriched pathways. (**F**) Gene Set Enrichment Analysis (GSEA) confirms positive enrichment of the p53 signaling pathway in blue light-exposed samples. (**G**) Venn diagram intersecting leading-edge genes from the p53 pathway with ferroptosis-associated gene sets identifies SLC7A11 as a key intersection. (**H**) Protein–protein interaction network shows interactions between p53 and ferroptosis-related proteins.

To delineate the molecular mechanisms orchestrating blue light-induced corneal epithelial ferroptosis, we performed RNA-sequencing analysis on HCE cells following 30 min blue light exposure. Principal component analysis demonstrated clear separation between control and blue light-exposed samples along the first principal component (Figure 4A). Sample correlation analysis revealed high within-group correlation coefficients, confirming experimental reliability and indicating that treatment effects predominated over biological variability (Figure 4B).

Differential expression analysis identified 901 genes significantly altered by blue light exposure (|log_2_FC| > 1, adjusted *p* < 0.05), comprising 579 upregulated and 322 downregulated genes, as visualized by a volcano plot (Figure 4C).

Gene Ontology enrichment analysis revealed the most significantly altered dimensions across three functional categories: biological process, cellular component, and molecular function (Figure 4D). Within the biological process category, differentially expressed genes were significantly enriched in cellular process, metabolic process, response to stimulus, and cell killing. In the cellular component category, alterations were predominantly associated with cell part, organelle, and cell junction-related components, providing a structural basis for the disruption of corneal barrier function induced by blue light exposure. Furthermore, molecular function analysis indicated a loss of antioxidant activity.

The Kyoto Encyclopedia of Genes and Genomes pathway enrichment analysis pinpointed specific signaling pathways modulated by blue light. Most significantly, we observed robust enrichment of the p53 pathway and ferroptosis pathway among the differentially expressed genes (Figure 4E). This coordinated enrichment of two intimately connected pathways suggested that p53 activation might represent the upstream regulatory event driving ferroptotic gene expression changes.

Given that conventional enrichment analysis considers only genes meeting significance thresholds, we performed Gene Set Enrichment Analysis (GSEA) to examine global transcriptional trends. GSEA confirmed significant positive enrichment of the p53 signaling pathway in blue light-exposed samples compared with controls (Figure 4F), validating that p53 pathway activation represents a genome-wide transcriptional program rather than an artifact of threshold-based selection. This systems-level confirmation establishes p53 signaling as a dominant response to blue light stress in corneal epithelial cells.

To identify mechanistic links between p53 activation and ferroptosis execution, we performed Venn diagram analysis to intersect the leading-edge genes of the p53 pathway identified from the GSEA with ferroptosis-associated gene sets. This analysis identified several genes common to both gene sets, most notably SLC7A11 and HMOX1 (Figure 4G). Given the central role of SLC7A11 in maintaining GSH synthesis and GPX4 activity, and its established regulation by p53, we selected SLC7A11 as the primary candidate bridging p53 signaling to ferroptotic cell death.

Protein–protein interaction network analysis revealed extensive interactions among p53 and ferroptosis-regulatory proteins. The network centered on p53 demonstrated direct and indirect connections to SLC7A11, GPX4, and other ferroptosis executioners (Figure 4H). This interactome analysis provided a molecular framework explaining how p53 activation could orchestrate the coordinated suppression of antioxidant defenses and promotion of ferroptotic cell death observed in our experimental systems. Collectively, transcriptomic analysis established p53 signaling as the master regulator of blue light-induced corneal epithelial ferroptosis and identified SLC7A11 as a key downstream effector.

### 3.5. p53 Knockdown Ameliorates Blue Light-Induced Ferroptosis via SLC7A11/GPX4 Axis Restoration

**Figure 5:**
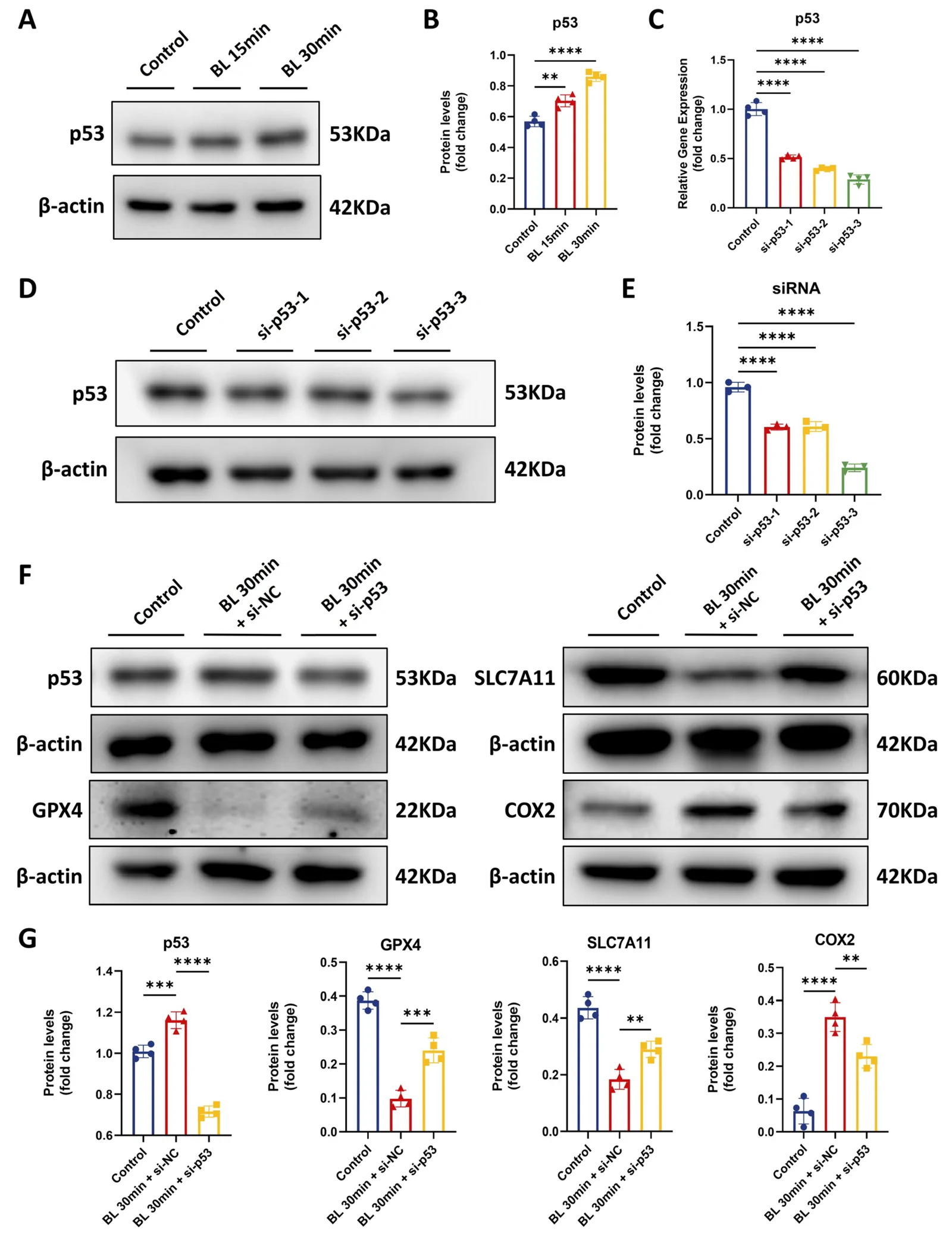
p53 knockdown attenuates blue light-induced ferroptosis via the SLC7A11/GPX4 axis. (**A**,**B**) Immunoblotting analysis confirms p53 protein upregulation after blue light exposure (*n* = 4). (**C**) qRT-PCR shows knockdown efficiency of three independent p53 siRNAs; si-p53-3 exhibits the highest efficacy (*n* = 4). (**D**,**E**) Immunoblotting validation confirms p53 protein knockdown by siRNAs (*n* = 3). (**F**,**G**) Immunoblotting analysis and densitometric quantification show that p53 knockdown reverses blue light-induced suppression of SLC7A11 and GPX4 and reduces COX2 upregulation (*n* = 4). Data are presented as mean ± SD. “**”, *p* < 0.01; “***”, *p* < 0.001; “****”, *p* < 0.0001. One-way ANOVA with Tukey’s post hoc test.

To functionally validate the role of p53 in blue light-induced ferroptosis, we employed siRNA-mediated gene silencing in HCE cells. First, we confirmed p53 activation following blue light exposure by immunoblotting. Compared with control cells, blue light exposure significantly increased p53 protein levels, confirming transcriptional findings at the protein level (Figure 5A,B).

We designed and screened three independent siRNA sequences targeting p53. qRT-PCR analysis demonstrated variable knockdown efficiencies, with si-p53-3 achieving the most effective mRNA reduction (Figure 5C). Immunoblotting confirmed superior protein-level knockdown with si-p53-3 (Figure 5D,E). This sequence was therefore selected for all subsequent experiments.

Having established an effective p53 knockdown model, we examined the effects on ferroptosis-regulatory proteins under blue light stress. As expected, blue light exposure in scrambled siRNA-transfected cells (BL 30 min + si-NC) significantly increased p53 protein levels, confirming successful stress induction (Figure 5F,G). Transfection with p53 siRNA prior to blue light exposure (BL 30min + si-p53) effectively suppressed this induction, demonstrating sustained knockdown efficacy under stress conditions.

Examination of ferroptosis regulators revealed that blue light exposure in control siRNA-transfected cells dramatically suppressed SLC7A11 and GPX4 protein levels. Remarkably, p53 knockdown significantly attenuated these reductions (Figure 5F,G). Consistent with these findings, the ferroptosis marker COX2, which increased following blue light exposure in control siRNA cells, was significantly reduced in p53-silenced cells.

These results demonstrate that p53 activation is required for blue light-induced suppression of the SLC7A11/GPX4 antioxidant axis and subsequent ferroptosis. Genetic inhibition of p53 preserves expression of these critical protective proteins, maintaining cellular capacity to counter oxidative stress and lipid peroxidation. The coordinated rescue of both SLC7A11 and GPX4 by p53 knockdown positions p53 as an upstream regulator coordinating multiple components of ferroptosis defense.

### 3.6. Topical p53 Inhibition Protects Against Blue Light-Induced Corneal Epithelial Damage In Vivo

Having established the mechanistic role of p53 in blue light-induced ferroptosis in vitro, we sought to evaluate whether pharmacological p53 inhibition could confer therapeutic benefit in a clinically relevant in vivo model. Mice subjected to 2-week blue light exposure received topical application of the specific p53 inhibitor PFT-α or vehicle control four times daily throughout the exposure period.

**Figure 6:**
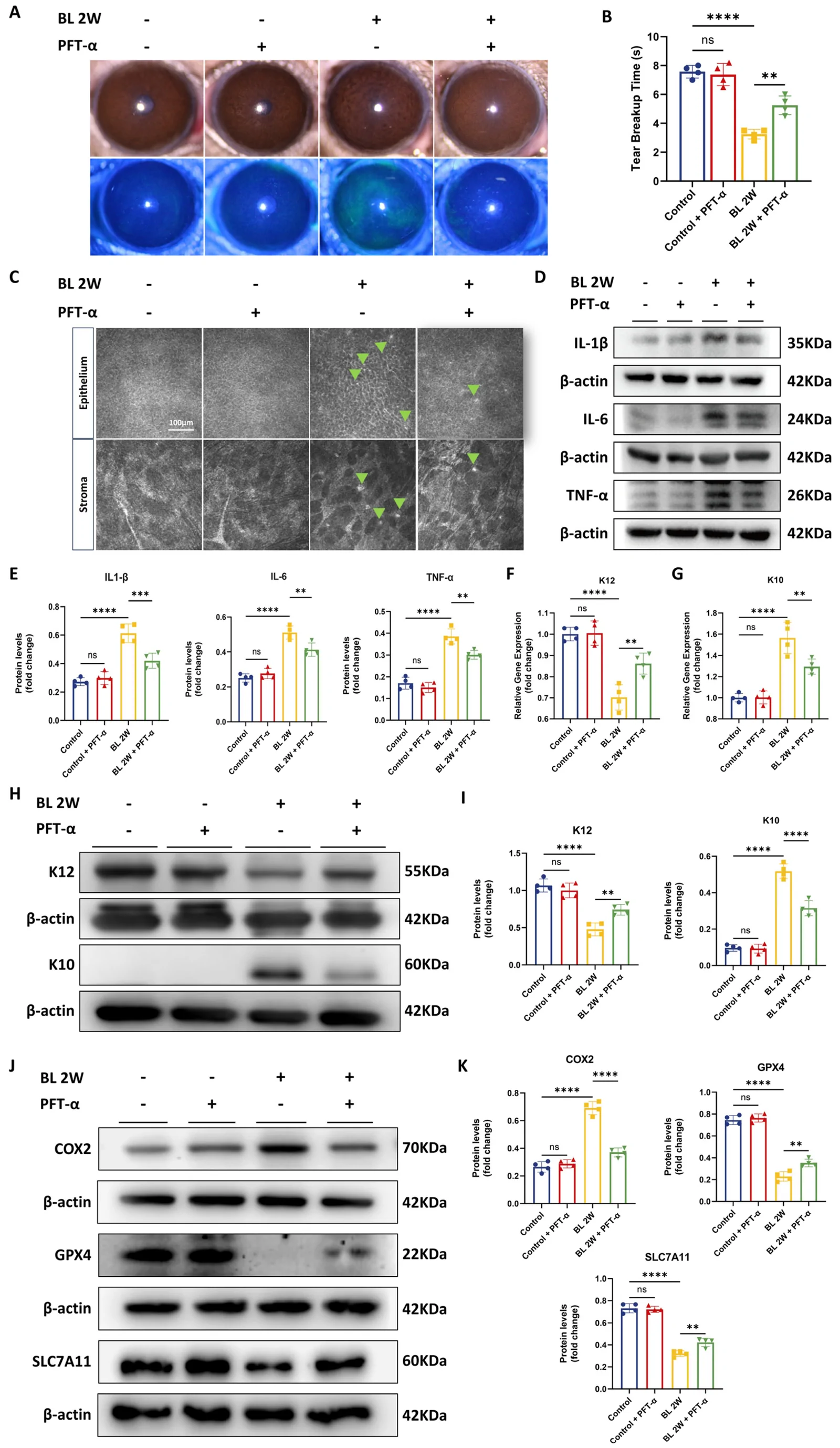
Topical p53 inhibition protects against blue light-induced corneal epithelial damage in vivo. Mice were subjected to 2 weeks of blue light exposure with topical application of p53 inhibitor pifithrin-α (PFT-α, 500 μM) or vehicle control four times daily. (**A**) Representative slit-lamp photographs after fluorescein staining show reduced corneal epithelial disruption in PFT-α-treated mice. (**B**) TBUT measurements demonstrate improved tear film stability with PFT-α treatment (*n* = 4). (**C**) In vivo confocal microscopy shows reduced inflammatory cell infiltration (green arrows) in PFT-α-treated corneas (scale bar = 100 μm). (**D**) Immunoblotting analysis of pro-inflammatory cytokines IL-1β, IL-6, and TNF-α in corneal tissues. (**E**) Densitometric quantification confirms that PFT-α treatment significantly attenuates blue light-induced upregulation of IL-1β, IL-6, and TNF-α (*n* = 4). (**F**,**G**) qRT-PCR shows that PFT-α treatment restores K12 mRNA levels and suppresses K10 mRNA levels (*n* = 4). (**H**,**I**) Immunoblotting analysis and quantification confirm corresponding protein changes (*n* = 4). (**J**,**K**) Immunoblotting analysis demonstrates that PFT-α treatment restores SLC7A11 and GPX4 expression and reduces COX2 levels in corneal tissues (*n* = 4). Data are presented as mean ± SD. “ns”, not significant; “**”, *p* < 0.01; “***”, *p* < 0.001; “****”, *p* < 0.0001. One-way ANOVA with Tukey’s post hoc test.

Ocular surface evaluation with fluorescein staining revealed substantial protection by PFT-α treatment. While vehicle-treated blue light-exposed mice exhibited extensive corneal epithelial staining, indicating severe barrier disruption, PFT-α-treated animals showed markedly reduced staining intensity (Figure 6A). Correspondingly, tear film stability assessment revealed that PFT-α treatment significantly prolonged TBUT (Figure 6B), indicating functional preservation of the ocular surface.

In vivo confocal microscopy demonstrated that PFT-α treatment attenuated blue light-induced inflammatory infiltration. Vehicle-treated blue light-exposed corneas displayed numerous hyperreflective inflammatory cells within the basal epithelium and stroma. In contrast, PFT-α-treated corneas showed substantially reduced inflammatory cell density (Figure 6C). This observation suggests that p53 inhibition not only preserves epithelial integrity but also limits secondary inflammatory responses to phototoxic damage. To further validate the anti-inflammatory effect of PFT-α at the molecular level, we examined the protein levels of pro-inflammatory cytokines IL-1β, IL-6, and TNF-α in corneal tissues by immunoblotting. As shown in Figure 6D,E, the blue light-induced upregulation of these inflammatory mediators was markedly attenuated by topical PFT-α treatment, consistent with the reduced inflammatory cell infiltration observed by confocal microscopy.

qRT-PCR analysis revealed that 2-week blue light exposure significantly reduced cornea-specific marker K12 mRNA levels while increasing squamous metaplasia marker K10 mRNA levels. Topical PFT-α treatment significantly reversed these alterations, restoring K12 and reducing K10 (Figure 6F,G). Immunoblotting confirmed these findings at the protein level (Figure 6H,I), establishing that p53 inhibition preserves corneal epithelial identity under blue light stress.

Consistent with our in vitro findings, blue light exposure dramatically reduced SLC7A11 and GPX4 protein levels. PFT-α treatment significantly attenuated these reductions, restoring SLC7A11 and GPX4. Correspondingly, the ferroptosis marker COX2, which increased following blue light exposure, was reduced by PFT-α treatment (Figure 6J,K). These in vivo results validate our mechanistic model and demonstrate that pharmacological p53 inhibition represents a viable therapeutic strategy for blue light-induced corneal epithelial damage.

## 4. Discussion

The present investigation provides comprehensive mechanistic evidence establishing blue light-induced corneal epithelial ferroptosis as a pathogenic mechanism in dry eye disease and identifying p53-mediated SLC7A11/GPX4 axis suppression as the central regulatory pathway. Using an in vivo mouse model, we demonstrate that blue light exposure triggers time-dependent corneal epithelial disruption characterized by barrier dysfunction, inflammatory infiltration, and epithelial thinning without affecting overall corneal thickness or transparency. At the cellular level, blue light induces ferroptotic cell death with hallmark features including intracellular iron accumulation, ROS generation, SLC7A11/GPX4 downregulation, and COX2 upregulation, accompanied by G1 cell-cycle arrest and pathological keratin expression switching. Transcriptomic analysis revealed p53 signaling pathway activation as the master regulator coordinating these ferroptotic changes, with genetic silencing and pharmacological inhibition confirming p53 as a therapeutic target. These findings advance our understanding of environmental light pollution as a contributor to dry eye and establish a mechanistic framework for developing targeted interventions.

The relevance of blue light as an environmental stressor has intensified with technological advancement and changing work patterns. Modern lifestyles increasingly involve prolonged exposure to digital displays, with average daily screen time exceeding 7 h [19]. While previous research has focused predominantly on retinal phototoxicity, the cornea’s position as the primary refractive interface necessitates direct exposure to incident light, making it a principal target for photochemical damage. Our findings demonstrate that even at intensities approximating typical display luminance (1000 lux), cumulative blue light exposure produces significant corneal pathology, suggesting that occupational and recreational screen use may contribute to the rising prevalence of dry eye symptoms reported in epidemiological studies [20].

The progressive nature of blue light-induced damage observed in our model has important implications for disease pathogenesis. Corneal epithelial disruption was detectable at 1 week and worsened progressively through 4 weeks, indicating cumulative effects rather than acute toxicity. This temporal pattern mirrors the insidious onset of dry eye symptoms in digital device users, where discomfort develops gradually over months to years of exposure [21]. The absence of corneal transparency changes suggests that early disease stages involve primarily epithelial dysfunction, potentially providing a window for intervention before irreversible structural changes occur. The inflammatory infiltration observed by confocal microscopy, increasing with exposure duration, likely represents secondary responses to epithelial damage and may contribute to the chronic inflammation characteristic of established dry eye disease [22].

While previous studies have implicated oxidative stress and apoptosis in blue light toxicity [7,16,17], our identification of ferroptosis as the primary cell death modality represents a significant advance in understanding ocular surface photodamage. The time-dependent accumulation of intracellular Fe^2+^ detected by FerroOrange staining provides direct evidence for iron dyshomeostasis, while the coordinated suppression of SLC7A11 and GPX4 identifies the molecular basis for antioxidant defense failure. The dramatic increase in ROS generation following blue light exposure, confirmed by both fluorescence microscopy and flow cytometry, represents the execution phase of ferroptosis.

The reciprocal regulation of SLC7A11 and COX2 observed in our study aligns with established ferroptosis signatures [10]. SLC7A11, as the light chain subunit of system Xc^−^, mediates cystine import essential for GSH synthesis. GPX4 subsequently utilizes GSH to reduce phospholipid hydroperoxides, breaking the chain reaction of lipid peroxidation. Consistent with this, a recent study demonstrated that blue light exposure downregulates GPX4 and SLC7A11, thereby inducing ferroptosis in conjunctival epithelial cells [23]. The coordinated downregulation of both proteins following blue light exposure thus creates a permissive environment for ferroptosis through dual mechanisms: impaired GSH synthesis and reduced capacity to neutralize existing lipid peroxides. COX2 upregulation, while not directly executing ferroptosis, serves as a reliable transcriptional marker and may contribute to inflammatory sequelae through prostaglandin production.

The keratin expression alterations we observed provide insight into the functional consequences of blue light exposure beyond cell death. K12 downregulation indicates loss of corneal epithelial differentiation, as K12 is essential for maintaining the specialized phenotype required for transparency and barrier function [24]. The reciprocal K10 upregulation suggests pathological transdifferentiation toward epidermal-type epithelium, a process termed squamous metaplasia observed in various ocular surface diseases including severe dry eye [25]. This phenotypic switch likely contributes to functional impairment through altered cytoskeletal organization, modified cell–cell adhesion, and compromised barrier properties. The partial reversal of these keratin changes by p53 inhibition suggests that differentiation alterations may be linked to the ferroptotic program rather than representing independent effects.

The transcriptomic analysis, providing the mechanistic framework for our study, revealed p53 signaling as the dominant pathway activated by blue light exposure. p53 has emerged as a central node integrating diverse stress signals and coordinating appropriate cellular responses ranging from cell-cycle arrest to senescence [13]. The enrichment of p53 pathway genes in our RNA-seq data, confirmed by GSEA demonstrating genome-wide activation, establishes blue light as a potent p53 agonist in corneal epithelial cells. This finding aligns with the established role of p53 in responding to DNA damage and oxidative stress, both of which are likely consequences of blue light exposure through direct photochemical effects and indirect ROS generation [13]. Our data demonstrate that in the context of blue light stress, p53 functions as a ferroptosis promoter through transcriptional suppression of SLC7A11. This finding is consistent with studies showing that p53 can repress SLC7A11 expression through direct promoter binding, reducing cystine uptake and sensitizing cells to ferroptosis [14]. In corneal epithelial cells exposed to blue light, the effect of p53 activation is clearly pro-ferroptotic, as demonstrated by the protective effects of both genetic silencing and pharmacological inhibition.

The identification of SLC7A11 as the critical intersection point between p53 signaling and ferroptosis execution has important therapeutic implications. SLC7A11 represents a druggable target, and compounds that enhance its expression or activity could potentially confer protection against blue light damage. However, our finding that p53 inhibition simultaneously restores both SLC7A11 and GPX4 suggests that targeting the upstream regulator may be more efficacious than modulating individual downstream effectors.

The in vivo validation of p53 inhibition using topical PFT-α demonstrates translational potential. The significant preservation of corneal epithelial integrity, tear film stability, and appropriate keratin expression following PFT-α treatment establishes proof-of-concept that pharmacological targeting of this pathway is feasible and effective. The concurrent reduction in inflammatory infiltration suggests that p53 inhibition may also limit secondary inflammatory responses that perpetuate disease [26,27]. These findings are particularly encouraging given that PFT-α has been extensively studied in preclinical models and exhibits favorable safety profiles [27,28]. The topical route of administration provides direct delivery to target tissues while minimizing systemic exposure, an important consideration for chronic use in ocular surface conditions.

The relationship between our findings and previous work on blue light-induced conjunctival damage merits consideration. We previously demonstrated that blue light exposure damages conjunctival stem cells through cAMP-PKA-Pax6 signaling, leading to dry eye [1]. The current study extends these observations by identifying distinct mechanisms operating in the corneal epithelium, suggesting that blue light affects multiple ocular surface compartments through pathway-specific mechanisms. The convergence on dry eye pathogenesis through complementary damage to both corneal and conjunctival epithelia likely explains the robust and reproducible phenotype observed in exposed animals. Future studies should explore potential interactions between these pathways and whether combined therapeutic approaches targeting both mechanisms might provide additive or synergistic benefits.

Several limitations of the present study warrant acknowledgment. First, while the 1000 lux intensity employed approximates maximum display illuminance, real-world exposure involves variable intensities, spectral compositions, and temporal patterns that may influence biological effects. Second, our use of a single 460 nm wavelength, while providing experimental precision, does not fully replicate the complex spectral output of actual displays, which contain broader blue light emissions. Third, the 8 h daily exposure, while modeling occupational screen use, may not reflect patterns of intermittent exposure characteristic of casual device use. Fourth, our studies focused on relatively short-term outcomes (up to 4 weeks), and the effects of long-term exposure remain to be determined. Fifth, while our in vivo confocal microscopy revealed inflammatory cell infiltration, we did not perform a systematic evaluation of corneal nerve morphology or density. Given that corneal nerve damage contributes to dry eye symptoms, this represents an important gap in our current understanding. Future investigations should examine whether blue light exposure induces structural or functional alterations in corneal nerves. Sixth, while we identified p53 as the master regulator, the upstream events linking blue light absorption to p53 activation require further elucidation. Seventh, we acknowledge that the HCE cell line used in this study is a virally transformed cell line, which may not fully recapitulate the physiology of primary corneal epithelial cells. Future investigations using primary cells or telomerase-immortalized cell lines are warranted to validate these findings in a more physiological context.

The clinical implications of our findings extend beyond immediate translation to therapeutic development. The demonstration that blue light at intensities encountered in daily life produces cumulative corneal damage suggests that public health interventions may be warranted. These could include engineering solutions such as blue light-filtering screens and eyewear, behavioral modifications, and policy measures establishing safety standards for display emissions. Future research directions should include clinical studies correlating digital device usage patterns with objective measures of ocular surface health, incorporating the mechanistic biomarkers identified in this study. Longitudinal studies examining whether blue light exposure accelerates age-related declines in ocular surface function would provide important public health guidance. Investigation of potential synergies between blue light exposure and other dry eye risk factors—including contact lens wear, hormonal status, and autoimmune conditions—could identify vulnerable populations. The development and optimization of p53-targeted therapies for ocular surface protection, including formulation development for enhanced corneal penetration and sustained release, represents a promising translational pathway. Additionally, exploration of nutritional interventions that support the SLC7A11/GPX4 axis, such as selenium supplementation or cystine-rich diets, might provide accessible preventive strategies.

The broader implications of our work extend to understanding how environmental factors shape ocular surface health in the modern world. The rapid technological changes in recent decades have outpaced our understanding of their biological consequences, particularly for organs like the eye that directly interface with the environment. The demonstration that a common environmental exposure—blue light from digital devices—can trigger specific molecular pathology through p53-mediated ferroptosis provides a framework for investigating other environmental stressors and their contributions to prevalent ocular diseases. This mechanistic understanding moves beyond descriptive associations to enable rational design of preventive and therapeutic interventions.

**Figure 7:**
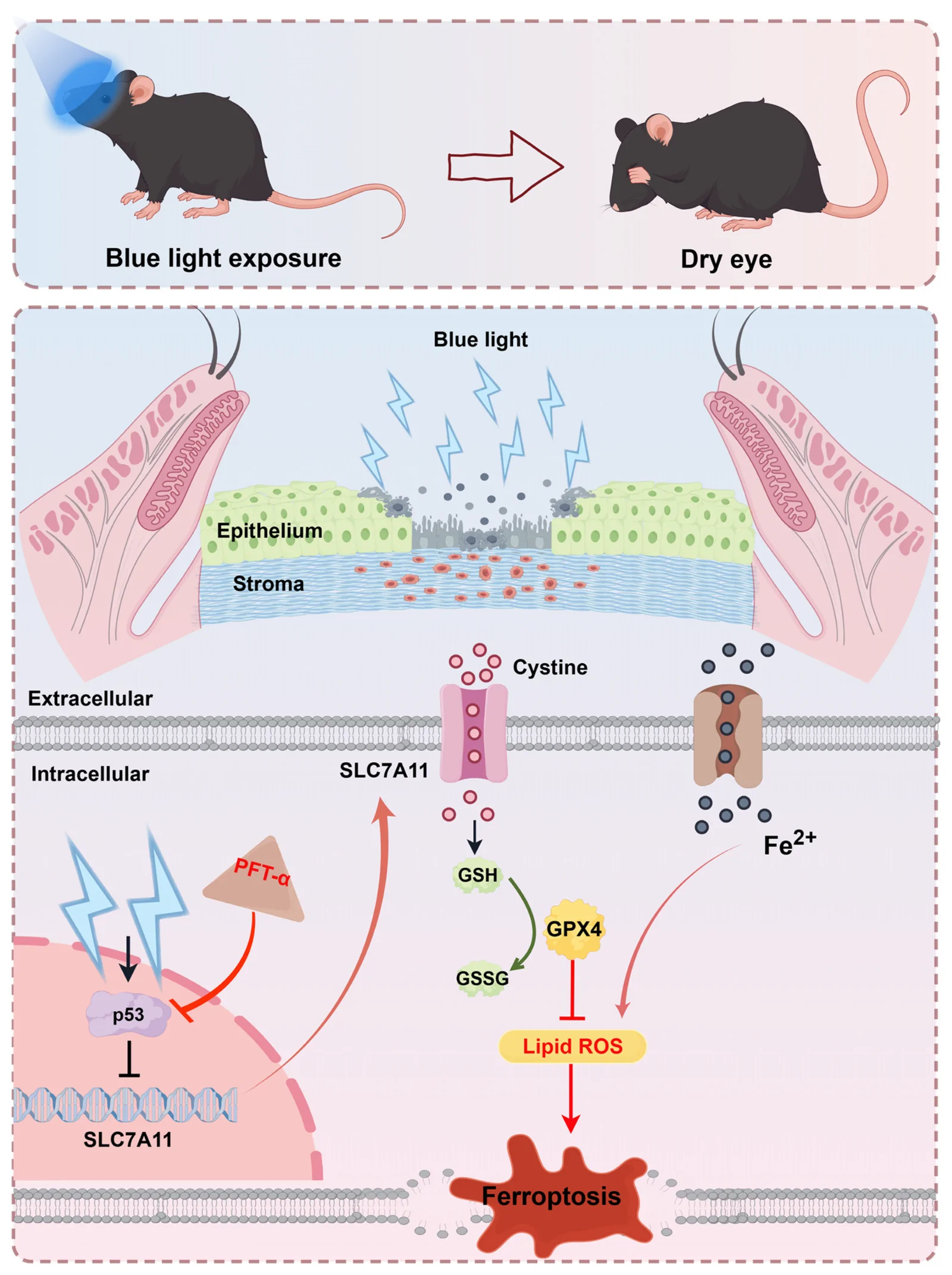
Integrated model of blue light-induced corneal epithelial ferroptosis.

This study establishes that blue light exposure induces corneal epithelial ferroptosis through p53-mediated suppression of the SLC7A11/GPX4 antioxidant axis, resulting in progressive ocular surface damage characteristic of dry eye disease. The protective effects of p53 inhibition in both in vitro and in vivo models identify a promising therapeutic strategy for blue light-associated ocular surface disorders. Based on our collective findings, we propose an integrated mechanistic model of blue light-induced corneal epithelial damage (Figure 7). Blue light exposure triggers p53 activation in corneal epithelial cells. Activated p53 transcriptionally suppresses SLC7A11 expression, which in turn reduces GPX4 levels, thereby triggering ferroptotic cell death. p53 activation also induces G1 cell-cycle arrest through suppression of CCND1 and CDK4, limiting regenerative capacity. Concurrently, blue light exposure promotes pathological keratin expression changes with downregulation of cornea-specific K12 and upregulation of squamous metaplasia marker K10. The net result of these coordinated molecular events is corneal epithelial thinning, barrier disruption, and inflammation characteristic of dry eye disease. Genetic silencing of p53 or pharmacological inhibition with PFT-α effectively interrupts this pathogenic cascade, preserving SLC7A11/GPX4 expression, maintaining epithelial identity, and protecting against blue light-induced ocular surface damage. These findings advance our understanding of environmental phototoxicity, provide a mechanistic basis for observed associations between digital device use and dry eye symptoms, and establish a foundation for developing targeted interventions to preserve ocular surface health in the digital age.

## 5. Conclusions

Blue light exposure induces progressive corneal epithelial damage characterized by barrier disruption, inflammatory infiltration, and epithelial thinning. The underlying mechanism involves p53-mediated ferroptosis through transcriptional suppression of the SLC7A11/GPX4 antioxidant axis, accompanied by G1 cell-cycle arrest and pathological keratin expression switching from K12 to K10. Genetic silencing of p53 or pharmacological inhibition with topical PFT-α effectively attenuates blue light-induced damage by restoring SLC7A11/GPX4 expression, preserving corneal epithelial identity, and maintaining ocular surface integrity. These findings establish p53-mediated ferroptosis as a central mechanism in blue light-associated ocular surface disease and identify p53 inhibition as a promising therapeutic strategy for preventing and treating dry eye related to digital device use.

## Figures and Tables

**Table 1:** Primer sequences for quantitative Real-Time PCR.

Gene	Forward Sequence (5′-3′)	Reverse Sequence (3′-5′)
CDK-4	ATGGAGGAGGAGGTGGAGGAG	CAGCCGGACAACATTGGGATG
CCND1	CGCCCTCGGTGTCCTACTTC	GACCTCCTCCTCGCACTTCTG
SLC7A11	CTCCTGCTTTGGCTCCATGA	CAGCTGGTAGAGGAGTGTGC
GPX4	CAGTGAGGCAAGACCGAAGT	CCGAACTGGTTACACGGGAA
K10	GACAAAGTTCGGGCTCTGGA	CCCCTGATGTGAGTTGCCAT
K12	GGAAATGCCCAGCTCCTCTT	GATCTGCATCTCCAGGTCGG
COX2	ATGATTGCCCGACTCCCTTG	CATGTTTGAGCCCTGGGGAT
p53	ATGAGCCGCCTGAGGTTGG	CAGTGTGATGATGGTGAGGATGG
β-actin	GTGCTATGTTGCCCTGGACTTC	ATGGAGGAGGAGGTGGAGGAG

## Data Availability

The original contributions presented in this study are included in the article. Further inquiries can be directed to the corresponding author.

## Abbreviations

The following abbreviations are used in this manuscript:

HCE	Human corneal epithelial
PFT-α	Pifithrin-α
Fe^2+^	Ferrous iron
ROS	Reactive oxygen species
K12	Keratin 12
K10	Keratin 10
GSH	Glutathione
SLC7A11	Solute carrier family 7 member 11
SLC3A2	Solute carrier family 3 member 2
GPX4	Glutathione peroxidase 4
COX2	Cyclooxygenase-2
CCK-8	Cell Counting Kit-8
TBUT	Tear break-up time
qRT-PCR	Quantitative Real-Time PCR
PI	Propidium iodide
SD	Standard deviation
IL	Interleukin
TNF	Tumor necrosis factor
CCND1	Cyclin D1
CDK4	Cyclin-dependent kinase 4
GSEA	Gene Set Enrichment Analysis
